# Optical Coherence Tomography in Patients with Chronic Migraine: Literature Review and Update

**DOI:** 10.3389/fneur.2017.00684

**Published:** 2017-12-13

**Authors:** Francisco J. Ascaso, Sara Marco, Javier Mateo, Mireya Martínez, Olivia Esteban, Andrzej Grzybowski

**Affiliations:** ^1^Department of Ophthalmology, Hospital Clínico Universitario Lozano Blesa, Zaragoza, Spain; ^2^Instituto de Investigación Sanitaria de Aragón (IIS Aragon), Zaragoza, Spain; ^3^Department of Ophthalmology, Poznan City Hospital, Poznan, Poland; ^4^University of Warmia and Mazury, Olsztyn, Poland

**Keywords:** migraine, optical coherence tomography, retinal nerve fiber layer, retina, choroid

## Abstract

Migraine is a chronic disease characterized by unilateral, pulsating, and often moderate-to-severe recurrent episodes of headache with nausea and vomiting. It affects approximately 15% of the general population, yet the underlying pathophysiological mechanisms are not fully understood. Optical coherence tomography (OCT) is a safe and reproducible diagnostic technique that utilizes infrared wavelengths and has a sensitivity of 8–10 μm. It can be used to measure thinning of the retinal nerve fiber layer (RNFL) in some neurological disorders. Although ophthalmologists are often the first specialists to examine patients with migraine, few studies have addressed the involvement of the optic nerve and retino-choroidal structures in this group. We reviewed the literature on the etiological and pathological mechanisms of migraine and the relationship between recurrent constriction of cerebral and retrobulbar vessels and ischemic damage to the optic nerve, retina, and choroid. We also assessed the role of OCT for measuring peripapillary RNFL thickness and macular and choroidal changes in migraine patients. There is considerable evidence of cerebral and retrobulbar vascular involvement in the etiology of migraine. Transitory and recurrent constriction of the retinal and ciliary arteries may cause ischemic damage to the optic nerve, retina, and choroid in patients with migraine. OCT to assess the thickness of the peripapillary RNFL, macula, and choroid might increase our understanding of the pathophysiology of migraine and facilitate diagnosis of retino-choroidal compromise and follow-up of therapy in migraine patients. Future studies should determine the usefulness of OCT findings as a biomarker of migraine.

## Introduction

Migraine is a chronic disease characterized by unilateral, pulsating, and often moderate-to-severe repetitive episodes of headache and vegetative symptoms such as nausea, vomiting, and extreme sensitivity to light and sound (1). This condition affects approximately 15% of the general population and is the most prevalent neurological disorder and the third most frequent global disorder in both genders (2). It mainly affects women aged 20–45 years (3, 4), and symptoms typically last 4–72 h (5–7).

Episodic migraine is defined as 0–14 headache days per month, whereas chronic migraine (CM) is defined as 15 or more headache days per month. Episodic migraine is classified into two major groups: migraine with aura (MwA, or classic migraine) and migraine without aura (MwoA, or common migraine) (8). Aura, which manifests as transient ocular and neurological disturbances, including flashes of light, blind spots, periorbital pain, and photophobia, affects about 25% of patients, usually immediately before but sometimes following the headache (9, 10).

Clinical data support ocular involvement in migraine. Thus, visual field compromise resembling that described in glaucoma has been reported (11). Perimetric damage progressed more quickly in patients with normal-tension glaucoma (NTG) and headache than that in individuals with NTG and no migraine (12). Several authors have explored visual acuity and visual field testing in migraineurs, although they did not observe any differences between migraineurs and controls (13, 14). Koban et al. (15) studied intraocular pressure (IOP) and biometric parameters and found no relevant differences between migraineurs during the attack and the healthy controls.

### Optical Coherence Tomography (OCT), a Promising Technology in Neurology

Decreased thickness of the retinal nerve fiber layer (RNFL) has been reported in several neurological disorders, such as multiple sclerosis, Alzheimer disease, and Parkinson disease (16–18). Ganglion cell layer (GCL) thickness could become a more appropriate morphological biomarker of axonal damage than RNFL in specific optic neuropathies (19, 20). OCT is a reliable, reproducible, objective, non-invasive, transpupillary diagnostic technique that enables quantitative *in vivo* high-resolution measurement of peripapillary RNFL, GCL, and choroid layer thickness (21–23). It generates a retinal cross-sectional map accurate to 5 µm. RNFL thickness is currently measured using OCT to assess glaucoma and maculopathies (24, 25). Decreased RNFL thickness has also been demonstrated by OCT in certain neurological disorders (26–30). In the last few years, several authors have used OCT to study whether the retina and choroid are involved in patients with migraine through the analysis of RNFL and macular and choroidal changes (Table 1).

**Table 1:** Published studies on optical coherence tomography (OCT) in migraine patients.

Reference	No of migraine patients/HC	OCT type	OCT measurements	Type of study	Results
Martinez et al. (32)	70/53	TD-OCT	RNFL thickness	Case–control	Thinner RNFL in the temporal sector in migraine patients. Migraine severity is associated with RNFL measurements
Sorkhabi et al. (57)	60/30	TD-OCT	RNFL thickness	Case–control	RNFL thinner in nasal quadrant in migraineurs
Kirbas et al. (58)	49 CM/40 HC	SD-OCT	RNFL and FT	Case–control	Superior quadrant RNFL thickness was thinner in CM patients. No macular changes in CM
Gipponi et al. (59)	24/16	SD-OCT	RNFL thickness	Case–control	Reduced RNFL thickness in the superior retinal quadrant in migraine patients. No influence of illness duration or frequency. No changes in macular parameters were found
Ekinci et al. (64)	90 (45 MwA, 45 MwoA)/30	SD-OCT	RNFL, GCL, and CT	Case–control	Reduced RNFL, GCL, and CT in individuals with MwA
Yülek et al. (60)	50 (30 MwA, 20 MwoA)/50	SD-OCT	RNFL, GCL, and FT	Case–control	Average RNFL thickness in migraine patients thinner than in HC. No correlation between length of migraine history and RNFL thickness
Demircan et al. (13)	76 (36 MwA, 40 MwoA)/38	SD-OCT	RNFL and CT	Case–control	Decreased thickness of RNFL in nasal sectors and foveal CT in migraineurs. Macular and choroidal thicknesses were not significantly different between the MwA and MwoA subgroups
Simsek (62)	40 (with and without WML)/40	SD-OCT	RNFL, GCL, and CT	Case–control	Diminished RNFL thickness in migraine patients with WML
Gunes et al. (63)	58/58	SD-OCT	RNFL	Case–control	Thinner RNFL thickness on the side of headache and on the contralateral side compared with control eyes
Colak et al. (16)	45/45	SD-OCT	RNFL, GCL, and CT	Case–control	Reduced RNFL in the superior and inferior quadrants of migraineurs compared to HC. Subfoveal, temporal, and nasal CT lower in the migraine group than in HC
Feng et al. (66)	432/281	SD-OCT	RNFL	Meta-analysis	Decreased RNFL thickness in the migraine patients
Cankaya and Tecellioglu (14)	39 (28 MwA, 40 MwA)/40	SD-OCT	RNFL	Case–control	Reduced FT values in migraine with aura
Reggio et al. (61)	72 (12 MwA, 21 MwoA, 44 CM)/42	SD-OCT	RNFL, GCL, FT, CT, and TMV	Case–control	Thinner RNFL, GCL, and CT in migraineurs, especially in CM. RNFL thickness is inversely associated with the frequency of migraine attacks
Acer et al. (82)	38 MwoA/44	SD-OCT	RNFL, GCL, and FT	Case–control	Sectorial RNFL thinning in MwoA patients. No significant changes in the macular area in MwoA

*CM, chronic migraine; MwA, migraine with aura; MwoA, migraine without aura; HC, healthy controls; TD-OCT, time domain optical coherence tomography; SD-OCT, spectral domain optical coherence tomography; RNFL, retinal nerve fiber layer thickness; GCL, ganglion cell layer thickness; FT, foveal thickness; TMV, total macular volume; CT, choroidal thickness; WML, white matter lesions detected using magnetic resonance imaging*.

### Vascular Involvement in Migraine

Previous studies suggested that brain hypoperfusion is common in MwA (31, 32), probably because of hypercoagulability and altered endothelial and vascular smooth muscle (33). Although the underlying pathophysiological mechanisms of migraine are not fully understood and many hypotheses have been proposed (31, 34–37), there is increasing evidence that the neurovascular system is involved (10, 31–40). Migraine attacks affect the trigeminal vascular system (TGVS), including the network of intra- and extracranial meningeal blood vessels and ocular structures (41, 42), and regulate vascular tone and the transmission of pain signals (43). Following activation of the TGVS, vasoactive neurotransmitters are released from peripheral terminations of the trigeminal nerve, causing vascular and inflammatory changes that result in pain (44). Migraine typically occurs when cerebral blood vessels undergo dramatic changes in caliber (45). The reduction in blood flow—due to the transient cerebral vasospasm that emerges prior to or during pain—is often limited to the posterior area of one hemisphere, leading to cerebral hypoperfusion (46). Alternating episodes of hyperperfusion and hypoperfusion during migraine may be confined to or start in other brain regions or even outside the brain, for example, in the retina and choroid. Even though constriction of cerebral and retrobulbar arteries is a transitory event, the chronic course with repetitive attacks could lead to permanent cerebral and retinal damage (47). In fact, retinal ischemia secondary to retinal artery occlusions has been described in patients with migraine (35, 48–54). Likewise, migraine is a recognized risk factor for ischemic optic neuropathy and NTG (55).

Visual symptoms resulting from occipital cortex involvement are more common than compromise of retinal or choroidal circulation. Therefore, in patients with these symptoms, OCT changes indicate retrograde trans-synaptic neuronal degeneration (RTSD) of the retinal ganglion cells (RGCs).

Although ophthalmologists are often the first specialists to explore patients with migraine, few studies have addressed the involvement of the optic nerve, retina, and choroid in this population.

This update reviews the literature on the etiological and pathological mechanisms of migraine and the relationship between recurrent constriction of cerebral and retrobulbar vessels and ischemic damage to the optic nerve, retina, and choroid in affected patients. It also provides evidence in favor of OCT for measurement of RNFL thickness and macular and choroidal changes in the different variants of migraine, which could be a biomarker of RTSD of the RGCs triggered by a lesion located on the posterior visual pathway.

### Methods

We searched PubMed for articles on the usefulness of OCT in patients with CM over a period of 25 years. We identified prospective and retrospective studies and case reports based on the following key words: *migraine, optical coherence tomography, OCT, retinal nerve fiber layer, RNFL, retina, choroid, etiology*, and *physiopathology*. We selected only articles written in English.

## Optic Nerve Structural Changes in Migraine

Compromised choroidal blood flow can produce focal ischemic damage in the optic disk (55). By using scanning laser polarimetry in patients with migraine (both with and without aura), Tan et al. (37) reported that migraine had no effect on RNFL.

The latest technological developments have made it possible to assess whether the retina is compromised in patients with migraine by using OCT to measure RNFL (Table 1). Decreased RNFL thickness reflects a reduced number of axons in these patients (32). However, results have not been very consistent. Thus, whereas some authors observed that mean peripapillary RNFL thickness was thinner in migraine patients than in healthy controls, others reported only a thinner RNFL in a specific quadrant (16, 18, 32, 56–62). This selective RNFL involvement might be associated with differences in the vulnerability of retinal axons to ischemia and with focal perimetric changes (63). The suspected axonal damage necessitates monitoring of RNFL thickness and visual field testing in migraine patients (62).

Ekinci et al. (64) studied subgroups of MwA and MwoA patients and demonstrated thinner RNFL in all sectors in MwA, but no significant differences in MwoA. The posterior area of a single brain hemisphere usually shows cerebral hypoperfusion during the attack (32). Thus, RNFL parameters may be more altered in MwA than in MwoA (63).

As migraineurs experience headaches almost always on the same side, several authors have studied unilateral involvement. In their investigation of the association between laterality of migraine and RNFL thickness in one-sided headache, Gunes et al. (63) reported a thin RNFL in patients with migraine compared with healthy controls and found that even though thinning was more relevant on the same side of the headache, the asymmetry was not statistically significant. The possibility of more relevant thinning of RNFL on the headache side could be secondary to lateralized permanent cortical changes, indicating that the hemisphere on the side of pain is more altered. Reduced blood flow has also been demonstrated in the brain on the affected side during attacks (62). Moreover, Hougaard et al. (65) explored unilateral headaches and investigated the connection between MwA and permanent gray matter anomalies. Using magnetic resonance imaging, Simsek (62) observed that in migraine patients with white matter lesions (WML), RNFL was thinner than in controls and migraine patients with no WML. Further research should analyze the relationship between brain WML and retinal damage to clarify whether there is a common pathogenic mechanism.

As for the frequency of migraine, some authors (18, 59) did not find any correlation with the RNFL thickness, whereas others reported an inverse correlation between RNFL thickness and the total number of monthly migraine attacks (61).

Peripapillary RNFL thickness measurements could also be associated with the length of migraine history. However, whereas some authors (18, 59) found no correlation between RNFL thickness and length of migraine history, Feng et al. (66) reported significant changes in RNFL based on the length of the history of migraine. Thus, mean RNFL thickness was decreased when migraine history was longer than 15 years. While controversial, this finding could be associated with glaucoma. In fact, Phelps and Corbett (67) observed a higher incidence of low-tension glaucoma in migraineurs. The origin of migraine is associated with the constriction of cerebral and retrobulbar arteries (18). During a migraine attack, vasospasm and reduced blood perfusion are typically demonstrated in a single hemisphere, although other cerebral regions and even retinal layers may also be altered by hypoperfusion (50). Migraine episodes may be linked to diminished blood flow in the retina and optic nerve, leading to irregular ocular perfusion and therefore to ischemia, which are implicated in the progression of glaucoma (32). The chronic nature of migraine, which is characterized by recurrent vasospasms and focal ischemia during attacks, could explain structural optic nerve damage, with the subsequent reduction in peripapillary RNFL thickness (61).

Furthermore, RNFL thickness values may correlate with the severity of headache. An important relationship between mean RNFL thickness and the migraine disability assessment score has been reported (57, 68). Nevertheless, Yülek et al. (60) evaluated the severity of migraine using the visual analog scale (69) and observed that the score was not correlated with RNFL thickness. The discrepancy might be explained by the use of different scoring systems and the diversity and severity of the headache in the study groups (66).

Such discrepant results may be attributed to differences in methodology and sample size, ethnic variations, and the absence of standardized migraine characteristics, including severity, length of migraine history, and frequency of attacks (61). Feng et al. (66) recently performed a meta-analysis of published case–control studies to explore changes in RNFL thickness in migraine patients assessed using OCT and reported lower peripapillary RNFL in patients with migraine than in healthy subjects. Therefore, although unspecific, OCT-based measurements of RNFL in migraine patients could be a useful technique for the study of ocular compromise and for a better understanding of pathogenesis.

## Macular Changes in Migraine

Retrograde trans-synaptic neuronal degeneration of the RGCs secondary to damage to structures in the posterior visual pathway has been demonstrated in various settings. Segmentation of retinal layers by spectral-domain OCT has enabled a detailed study of functional areas. This is most useful at the macula, where the density of RGCs is maximal and where other anatomical structures are absent (70, 71). Several authors have used OCT to study possible changes in macular thickness in patients with migraine. Their results are contradictory because, although most found no change in either foveal or macular parameters in migraine patients (18, 59, 62), some reported that foveal thickness (FT) values (19) or GCL (61, 64) were thinner in MwA than in MwoA. The more diminished blood flow in MwA would explain this finding, given that in MwoA, at least between attacks, pulsatile choroidal blood flow is not compromised.

Therefore, altered choroidal blood flow can produce ischemic damage in retinal tissue, leading to photoreceptor dysfunction and eventually GC death (32, 72, 73). In fact, MwA patients seem to have a higher risk of ischemic events than MwoA (31, 68).

Finally, no correlation was observed between FT and disease duration/length or number of episodes (72).

## Choroidal Changes in Migraine

Choroidal vascular deficiency causes decreased choroidal thickness (CT), which results in dysfunction of retinal pigment epithelium and photoreceptors (74, 75). Choroid thickness may decrease as a consequence of decreased blood flow in retinal and ciliary vessels (31).

Today, it is possible to estimate CT using advanced OCT devices. However, major discrepancies have been observed. Thus, whereas some authors report an increase in CT during attacks, reflecting altered ocular circulation (76, 77), others report reduced CT during attacks (78, 79). Reggio et al. (61) recently reported that CT was thinner in migraineurs than in healthy individuals. Colak et al. (16) found that RNFL and CT were thinner in MwA than in healthy controls, which is secondary to an increasing loss of GC and axons. The disparity between authors could be explained by the fact that both types of migraine involve changes in blood perfusion during their course, albeit with different severity.

Ocular pulse amplitude (OPA) refers to the gap between systolic and diastolic IOP. Measured using dynamic contour tonometry, it is considered an indirect indicator of choroidal blood flow and reflects fluctuations in IOP related to the volume of blood pumped in the interior of the eye during the cardiac cycle (80, 81). Acer et al. (82) estimated choroidal flow by evaluating OPA and were unable to demonstrate relevant dissimilarities in OPA between patients with MwoA and controls, thus revealing that pulsatile choroidal blood flow is not always altered in these subjects. In fact, other studies had previously demonstrated choroidal and retinal involvement in patients with migraine (32, 59, 64).

Amaurosis fugax in migraineurs has been proposed as a sign of affected choroidal blood flow in patients with migraine (83), as have alterations in retinal pigment epithelium on fundoscopy after attacks (84).

## Conclusion

The aim of this update was to review the literature on the etiological and pathogenic mechanisms of migraine. We analyze evidence on the role of OCT for measurement of peripapillary RNFL thickness and macular and choroidal changes in migraineurs. We conclude that there is abundant evidence on cerebral and retrobulbar vascular involvement in the etiology of migraine. The transitory and recurrent constriction of the retinal and ciliary arteries may cause ischemic damage to the optic nerve, retina, and choroid in migraineurs. Although OCT-based studies show conflicting results, our data suggest that the retina and choroid are altered in patients with migraine, mainly CM. Moreover, RNFL parameters and FT seem to be more altered in MwA than in MwoA. RTSD of the RGCs and RNFL following injury to the occipital lobe have been reported in experimental studies. Magnetic resonance imaging and OCT could enable us to visualize and quantify RTSD and analyze its time course (85). Therefore, OCT-based measurement of peripapillary RNFL, GCL, and macular and CT might improve our understanding of the pathophysiology of migraine and facilitate diagnosis of retino-choroidal compromise and follow-up of the effectiveness of therapies in migraine patients. Future studies should determine the usefulness of OCT findings as a biomarker of migraine. Finally, migraine is such a heterogeneous disorder that it will be difficult to cluster patients in categories. Treatment (e.g., triptans, ergot derivatives) may also lead to vasoconstriction, which could—theoretically—bring about changes in OCT values.

## Author Contributions

FA, JM, SM, MM, OE, and AG: substantial contributions to the conception or design of the work; the acquisition, analysis, and interpretation of data for the work; FA, JM, SM, MM, OE, and AG: drafting the work or revising it critically for important intellectual content; FA, JM, SM, MM, OE, and AG: final approval of the version to be published; FA, JM, SM, MM, OE, and AG: agreement to be accountable for all aspects of the work in ensuring that questions related to the accuracy or integrity of any part of the work are appropriately investigated and resolved.

## Conflict of Interest Statement

The authors declare that the research was conducted in the absence of any commercial or financial relationships that could be construed as a potential conflict of interest.
